# Cross-cultural adaptation and validation of the Chinese version of Toronto Extremity Salvage Score for patients with extremity sarcoma

**DOI:** 10.1186/s40064-016-2818-9

**Published:** 2016-07-19

**Authors:** Leilei Xu, Minghui Sun, Weixiang Sun, Xiaodong Qin, Zezhang Zhu, Shoufeng Wang

**Affiliations:** Department of Orthopedic Surgery, The Affiliated Drum Tower Hospital of Nanjing University Medical School, Zhongshan Road 321, Nanjing, 210008 China

**Keywords:** TESS, Chinese, Extremity sarcoma, Function

## Abstract

**Objective:**

As a widely used instrument for patients with extremity sarcoma, the Toronto Extremity Salvage Score (TESS) has never been cross-culturally adapted for Chinese population. The objective of our study was to investigate the comprehensibility, reliability and validity of the Chinese version of TESS for use in patients with extremity sarcoma.

**Methods:**

A consensus version of the Chinese TESS was developed under the review of a committee according to international guidelines. 64 patients were recruited to complete the Chinese TESS, the Musculoskeletal Tumor Society (MSTS) Rating Scale, and the Quality of Life Questionnaire Core 30 (QLQ-C30). Reliability was assessed using the intra-class correlation coefficient (ICC) and Cronbach’s α. Validity was assessed with Pearson’s correlation between the similar domains of the two questionnaires.

**Results:**

The ICCs for the test–retest reliability was 0.932 for the upper extremity questionnaire and 0.893 for lower extremity questionnaire, respectively. The Cronbach’s α was 0.953 for the lower extremity questionnaire and 0.921 for the upper extremity questionnaire, respectively. Convergent validity of the TESS based on Pearson correlation coefficients indicated significantly moderate to high correlations between the TESS and the MSTS as well as the QLQ-C30, with r ranging from 0.535 to 0.782.

**Conclusions:**

The Chinese TESS is a comprehensible, reliable, and valid instrument that can be utilized for future cross-cultural international studies of extremity sarcoma.

## Background

Sarcoma is a rare type of cancer that represents approximately 1 % of all newly diagnosed cancers (Borden et al. 2003). Due to the significant impact of extremity sarcoma surgery on the patients’ function, health related quality of life (HRQoL) is now recognized as an important outcome measure of the surgery (Hoffmann et al. 2006; Griesser et al. 2012; Ruggieri et al. 2011). Development of instruments to measure HRQoL is essential to determine patients’ perceived physical and mental health (Schreiber et al. 2006; Lopez-Guerra et al. 2011). To be noted, patients with sarcoma may have a significant heterogeneity regarding tumor type, reconstructive techniques and the extent of tissue excised during surgery. Therefore, it is important to develop an instrument that takes heterogeneity into account when measuring functional outcome. And a disease-specific measure rather than a generic measure is preferred for the proper assessment of physical function in patients with extremity sarcoma.

To date, several disease-specific questionnaires have already been developed to evaluate functional outcomes for patients undergoing surgery for extremity sarcomas (Davis et al. 1996; Enneking et al. 1993; Bekkering et al. 2009). Some examiner-dependent clinical measures such as range of motion and muscle strength or a combination of symptoms and mobility have been used. However, measures that reflect the patient’s perception seems more desirable in clinical practice. As a patient-completed questionnaire that takes into account the heterogeneity of sarcomas, the Toronto Extremity Salvage Score (TESS) was developed for assessing physical function of patients undergoing surgery for extremity sarcoma (Davis et al. 1996).

The TESS questionnaire is a disease-specific and self-administered questionnaire based on the definitions of disability, impairment, and handicap as documented by the World Health Organization (Davis et al. 1996). The content of the TESS includes the types of functional difficulties experienced by extremity sarcoma patients, such as body movement, mobility, self-care, and performing daily tasks. To date, several studies have reported the validation of the TESS in a Portuguese (Saraiva et al. 2008), Danish (Saebye et al. 2014), Korean (Kim et al. 2015) and Japanese version (Ogura et al. 2015), yielding good test–retest reliability and internal consistency. However, to the best of our knowledge, there is no study that addresses the application of TESS to patients from the Chinese population. Herein, this study was conducted to investigate the comprehensibility, reliability and validity of the Chinese version of TESS for use in patients with extremity sarcoma.

## Methods

### Subjects recruitment

The protocol of recruitment of participants was approved by the ethics committee of the hospital. Patients who visited our clinic center between March 2011 and September 2014 were prospectively evaluated for the eligibility of recruitment in this study. The following inclusion criteria were used: (1) aged more than 18 years; (2) at least 1 year after curative surgery for histologically confirmed extremity sarcoma; (3) without local recurrence, distant metastasis or complications related to surgery. Finally, 64 patients including 35 male and 29 female participated in the study and gave their informed consent. Demographic data were collected from the medical records, including patients’ age, gender, location of the tumor, histological type, type of surgery and period of follow-up.

### Translation and adaptation of the TESS

The translation and adaptation processes of the TESS were performed according to the guidelines used by previous literatures (Saraiva et al. 2008; Kim et al. 2015; Ogura et al. 2015). The English version of the TESS was translated into Chinese independently by three native Chinese bilingual translators who were familiar with the topic and the research concept. Subsequently, the two translators with the medical background analyzed and compared the three translations together with two senior musculoskeletal oncologists, and then combined them into one single translation. The consensus version was back translated into English independently by two bilingual translators who were kept blind to the procedures of the forward translation. All versions of the translation were analyzed by the expert committee, which was comprised of the 5 translators, one methodologist, and 2 orthopedic surgeons (X.L. and W.S.). The committee evaluated conceptual equivalence of all items and answers, and discrepancies between members were discussed. The pre-final version of the TESS was created after consensus was reached among the expert committee. In the pre-test step, 40 volunteers aged more than 18 years were recruited from the local community to test the pre-final Chinese version of the TESS. These volunteers were questioned about their understanding of the questionnaire items. Most of the items in the questionnaire could be correctly understood and answered by the volunteers. There was a high incidence of “not applicable” with regard to the question on sexual activity, which was therefore omitted from the questionnaire. After this process, the final version of the Chinese version of the TESS was developed. It consists of a lower extremity and an upper extremity version both with 29 questions. Each question is rated on 5-point scale, including “impossible to do,” “extremely difficult,” “moderately difficult,” “a little bit difficult,” and “not at all difficult”. The participant can select “not applicable” when the question is not a usual activity. The total score ranges from 0 to 100, with higher scores indicating better function.

### Reliability and validity test of the TESS

All the patients were asked to answer the TESS questionnaire at their follow-up visit after surgery. The time needed for the completion of the questionnaire was recorded for each participant. To examine the test–retest reliability, patients were asked to complete the same TESS questionnaire 1 week later. All the patients returned the second questionnaire used for analysis. The data were collected by an author (S.W.) who was kept blind of the patients’ diagnosis or treatment.

To examine the convergent validity, the patients were also asked to complete the Musculoskeletal Tumor Society (MSTS) Rating Scale, and the Quality of Life Questionnaire Core 30 of European Organization for Research and Treatment of Cancer (EORTC QLQ-C30). The MSTS Rating Scale is a widely used functional score for patients with extremity sarcoma (Rebolledo et al. 2013). It consists of factors pertinent to the patient, including pain, function, emotional acceptance, use of any external support, walking ability, and gait alteration. A value of 0–5 points (maximum overall score, 30 points) was assigned to each of these factors. The QLQ-C30 is a 30-item questionnaire used to evaluate the function and QoL in cancer patients (Fitzsimmons et al. 1999). The following scales among the functional scales of QLQ-C30 were used in this study, including physical functioning (PF), role functioning (RF), social functioning (SF) and QoL. A high score on the functional scales represents a high level of QoL and a high level of functionality.

### Factor analysis

Confirmatory factor analysis was carried out with each factor being specified to load on its subscale. Model fit was assessed with the following parameters, including the comparative fit index (CFI), the normed fit index (NFI), root-mean square error of approximation (RMSEA), and the 90 % confidence intervals (CIs) of RMSEA. A good fit of the model was indicated by ratios between the Chi square test and degrees of freedom less than 3, CFI and NFI values not less than 0.90, and RMSEA not more than 0.08.

### Statistical analysis

SPSS for Windows version 16.0 statistical software (SPSS, Chicago, IL, USA) was used for statistical analyses. Descriptive demographic data and scores were reported as mean values ± standard deviation. The test–retest reliability was examined using intra-class correlation coefficient (ICC). The internal consistency was evaluated by Cronbach’s α, with a value >0.70 considered acceptable. To determine the convergent validity of the TESS, correlations between similar domains of the TESS and the QLQ-C30 or the MSTS were analyzed by Pearson correlation analysis. The correlation coefficient larger than 0.40 was considered to indicate adequate convergent validity.

## Results

The mean time used to complete the TESS questionnaire was 285.2 ± 75.7 s (range 182–382). The baseline characteristics of the patients were summarized in Table 1. The mean age at the time of completion of the questionnaire was 47.5 ± 15.2 years (range 18–68 years). The most common histological types were osteosarcoma (n = 15) and chondrosarcoma (n = 13) for bone sarcoma and liposarcoma (n = 11) for soft tissue sarcoma. Most patients received limb salvage surgery followed by chemotherapy with a mean follow-up of 1.9 ± 1.2 years (range 1–3 years).

**Table 1:** Baseline characteristics of the patients

Characteristic	Patients (n = 64)
Age (year)	47.5 ± 15.2
Gender	
Male	35
Female	29
Time from surgery (year)	1.9 ± 1.2
*Tumor location*	
Upper extremity	
Upper arm	12
Shoulder	5
Forearm/wrist	4
Elbow	2
Lower extremity	
Thigh	14
Lower leg	10
Pelvis/hip	7
Knee	6
Ankle/foot	4
*Histological type*	
Bone	
Osteosarcoma	15
Chondrosarcoma	13
Soft tissue	
Liposarcoma	11
Undifferentiated pleomorphic sarcoma	6
Myxofibrosarcoma	5
Fibrosarcoma	4
Leiomyosarcoma	3
Epithelioid	3
Others	4
*Surgery type*	
Amputation	11
Limb salvage	53

Results of reliability evaluation for Chinese version of the TESS were summarized in Table 2. The ICC for the test–retest reliability was 0.932 for the upper extremity questionnaires (95 % confidence interval (CI) = 0.745–0.971) and 0.893 for lower extremity questionnaires (95 %CI = 0.755–0.937), respectively. In addition, the test for internal consistency showed strong reliability with a Cronbach’s α of 0.953 for the lower extremity questionnaire and 0.921 for the upper extremity questionnaire. The Pearson correlation coefficients between one item and the total score ranged from 0.533 to 0.869 for the lower extremity questionnaire and from 0.587 to 0.913 for the upper extremity questionnaire.

**Table 2:** Reliability test of the simplified Chinese version of the TESS

The TESS	ICC (95 % CI)^a^	Cronbach’s α	Correlation^b^
Lower extremity	0.893 (0.755–0.937)	0.953	0.533–0.869
Upper extremity	0.932 (0.745–0.971)	0.921	0.587–0.913

^a^ICC indicates Intraclass correlation coefficient; 95 % CI indicates 95 % confidential interval

^b^Correlations between one item and the total score excluding that item using Spearman rank correlation coefficient

As shown in Table 3, the overall mean score of the TESS was 87.6 ± 20.2 (range 40–100). The mean scores of the lower extremity questionnaire and the upper extremity questionnaire were 83.4 (range 40–100) and 92.3 (range 45–100), respectively. The mean score of the MSTS was 24.6 (range 5–30). The mean score of the QLQ-C30 was 78.1 (range 0–100) for PF, 67.5 (range 0–100) for RF, 71.2 (range 0–100) for SF and 68.2 (range 0–100) for QoL, respectively.

**Table 3:** Patients’ score of the TESS, the MSTS and the QLQ-C30

	Lower extremity	Upper extremity
	(n = 41)	(n = 23)
TESS^a^	83.4 ± 19.5	92.3 ± 22.7
MSTS^b^	25.5 ± 6.5	23.1 ± 4.3
QLQ-C30^c^		
Physical functioning	77.4 ± 25.3	78.9 ± 18.4
Role functioning	66.3 ± 23.2	68.2 ± 21.5
Social functioning	72.1 ± 15.6	70.8 ± 20.3
Quality of life	69.3 ± 24.1	66.4 ± 26.2

^a^TESS indicates Toronto Extremity Salvage Score

^b^MSTS indicates Musculoskeletal Tumor Society

^c^QLQ-C30 indicates Quality of Life Questionnaire Core 30

Convergent validity of the TESS based on the comparison with the MSTS and the QLQ-C30 was shown in Table 4. Pearson correlation coefficients indicated significantly moderate to high correlations between the TESS and the MSTS or the 4 scales of QLQ-C30, with r ranging from 0.532 to 0.782. The strong correlations were observed between the TESS and the PF scale of QLQ-C30. Table 5 shows the results of the factorial analysis of the TESS. According to the pre-established thresholds of RMSEA, NFI and CFI, the 2-factor model met the criteria for a good fit.

**Table 4:** Convergent validity of the Chinese TESS

	Lower extremity (r)	Upper extremity (r)
MSTS	0.637^†^	0.682^†^
QLQ-C30		
Physical functioning	0.782^†^	0.735^†^
Role functioning	0.638^†^	0.547^†^
Social functioning	0.712^†^	0.532^†^
Quality of life	0.689^†^	0.635^†^

^†^p < 0.05

**Table 5:** Factor analysis of the Chinese TESS

Model	χ^2^/*df*	CFI	NFI	RMSEA	90 % CI
2-Factor model	1.79	0.92	0.91	0.068	0.056–0.079

*χ* ^*2*^ */df* ratio between the Chi square test and degrees of freedom, *CFI* comparative fit index, *NFI* normed fit index, *RMSEA* root-mean square error of approximation, *CI* confidence interval

## Discussion

The original TESS questionnaire was a valid, reliable and sensitive self-reported instrument to evaluate the functional outcome of sarcoma patients. To date, different language versions of the TESS have been validated in Western and Asian populations (Davis et al. 1996; Saraiva et al. 2008; Saebye et al. 2014; Kim et al. 2015; Ogura et al. 2015). For the first time, this study sought to validate the cross-culturally adapted Chinese version of the TESS. During our translation of the TESS into Chinese, cross-cultural bias was taken into account according to the guidelines reported by Guillemin et al. (Guillemin et al. 1993). A few cultural discrepancies were encountered and some items of the TESS were therefore modified accordingly. The question on sexual activity was omitted from the questionnaire due to the high incidence of “not applicable”. Comparably, there was also a high rate of “not applicable” regarding the question on sexual activity in the Korean and Japanese version of the TESS (Kim et al. 2015; Ogura et al. 2015). We believed that it might be attributed to the different cultural background between the Asian and Western populations. Besides, “cutting food while eating” was replaced by “using chopsticks when eating” as the majority of the Chinese use chopsticks at meals instead of a knife and fork. After translation and cross-cultural adaptation, the mean completion time of the TESS was short and comparable with results reported by other studies. Therefore, we confirmed that the Chinese version of the TESS could be clearly understood and easily administered to the patients.

Compared with other language versions of the TESS, the Chinese TESS showed similar test–retest reliability, internal consistency and validity. The ICCs of 0.93 for the upper extremity questionnaire and 0.89 for the lower extremity questionnaire demonstrated a high level of reliability. Comparably, Saebye et al. (2014) reported the ICC for the upper and lower extremity questionnaires in the Danish population were 0.96 and 0.88 (95 % CI 0.697–0.956), respectively. In a recent study performed in the Korean population, Kim et al. (2015) reported ICCs of 0.979 for the upper extremity TESS and 0.874 for the lower extremity TESS respectively. In our study, Cronbach’s α value for internal consistency was 0.953 and 0.921 for the lower and the upper extremity questionnaire, respectively. Similarly, the internal consistency of the lower and the upper extremity questionnaire reported in previously studies was 0.90 and 0.94 in Denmark and 0.978 and 0.989 in Korea (Saebye et al. 2014; Kim et al. 2015). To investigate the convergent validity, comparisons were made among the TESS, the MSTS and the functional part of the QLQ-C30. We chose the MSTS and QLQ-C30 as they are reliable questionnaires developed for cancer patients. Our results showed that there were good correlations between the TESS and the MSTS and between the TESS and the QLQ-C30. The Pearson correlation coefficients indicated significantly moderate to high correlations between the TESS and the other two questionnaires. Collectively, we believed that the current Chinese version of the TESS questionnaire could well maintain the properties of the original version to measure the QoL of sarcoma patients.

The TESS validation study reported here reflects the rising importance of expanding our knowledge on the physical function of extremity sarcoma patients. As indicated by the good correlation with the PF scale of QLQ-C30, the TESS can specifically evaluate variable levels of physical function. However, other important domains of QoL, including body image, mental status and social activities were not taken into account when applying the TESS to patients with musculoskeletal tumors. Design of such a disease-specific instrument that can evaluate HRQOL for patients with musculoskeletal tumors comprehensively is awaited.

Several limitations still exist in the current study. First, the present study did not test the ability of the TESS to detect the responsiveness of patients to the treatment, which was limited mainly by a relatively small number of patients. A larger sample size is therefore recommended for to clarify this result in future studies. Second, we did not investigate the relationship between the TESS scores and time from surgery or type of surgery. Herein, further investigation into the sensitivity of the TESS to detect the HRQOL in patients with different time from surgery or undergoing different types of surgery was warranted.

In conclusion, we have translated the TESS questionnaire into Chinese and validated the questionnaire. Our results showed that Chinese version of the TESS is a comprehensible, reliable, and valid instrument and can be utilized for future cross-cultural international studies of extremity sarcoma.
